# The barbed suture of deep fascia walks away and pierces through skin far from the incision – a case report

**DOI:** 10.1002/ccr3.821

**Published:** 2017-01-28

**Authors:** Shufeng Li, Huaqiang Sun, Zhaolong Yu, Wei Wang, Teng Wang, Xinfeng Yan

**Affiliations:** ^1^ Department of OrthopedicsQianfoshan HospitalShandong UniversityJinanShandongChina; ^2^ DICAT Biomedical Computation CentreVancouverBritish ColumbiaCanada

**Keywords:** Barbed suture, complication, total hip arthroplasty

## Abstract

Barbed suture has been widely used in surgeries. However, this technique may raise many problems. Here, we report that the barbed suture of deep fascia walks away and pierces through skin far from the incision in a 61‐year‐old male 6 weeks after total hip arthroplasty.

## Introduction

Barbed suture has been widely used in surgical incisions, especially in joint surgery and plastic surgery. This technique has its advantages, such as shortening operation time 1, 2, 3, 4, tightening the joint capsule more effectively 5, 6, 7, and decreasing scars 2, while the complication rate remains similar with conventional suturing methods 8, 9. However, barbed suture has generated new issues. It has been reported that skin suture may pierce through the incision 4, 10. Here, we encountered a case whose barbed suture of deep fascia ran away and pierced through the skin 20 cm away from the incision 6 weeks after the total hip arthroplasty (THA).

## Case Report

**Figure 1:**
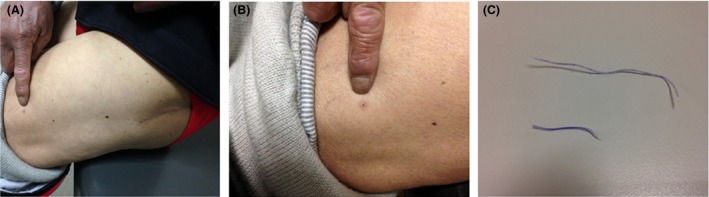
(A and B) One week after barbed suture piecing and the position of barbed suture piercing. (C) The barbed suture of piercing.

The patient was a 61‐year‐old male and received left THA surgery due to osteonecrosis of femoral head. During the surgery, continuously suturing the fascia and fat layer was performed using one biodegradable No.2 two‐way barbed suture (36 + 36 cm), and continuously suturing the skin was performed with one biodegradable No.2‐0 two‐way barbed suture. The incision healed well without swelling. The patient was discharged 5 days after surgery. Six weeks after the surgery, skin ulceration was found in the anterolateral thigh, about 20 cm away from the incision (Fig. 1A and B), and the suture was seen piercing out from the ulceration. The patient did not feel pain or any other symptom during the barbed suture's walking. The patient pulled out the suture at home, which was found the No.2 biodegradable bidirectional barbed suture for deep fascia. It was about 10 cm long and broke into two parts during pulling out (Fig. 1C). The ulcer healed quickly after the suture was pulled out. The barber suture of the skin did not walk away. At present, it has been 9 months after the THA, and the patient's left hip functions well without infection, pain, or other symptoms. The study was approved by the review board of Qianfoshan Hospital, and written consent form was obtained from the patient.

## Discussion

The emergence of barbed suture is a revolutionary progress for surgical incision. The use of barbed suture has become increasingly popular. However, its efficacy and safety continue to be debated. There are reports about skin suture piercing through the incision 4, 10. However, deep fascia sutures piercing through skin far away from the incision location have not been reported.

Barbed suture uses biodegradable material. It is a type of knotless surgical suture that has barbs on its surface. While suturing tissue, these barbs penetrate inside the tissue and lock them into one direction, eliminating the need for knots to tie the suture. The directions of barbs along two ends of suture are opposite; therefore, under normal circumstances, the two ends restraint each other to avoid suture running 1. But when suture breaks in one point because of biodegradation absorption, one end of barbed suture will run in certain direction with the tissue movement. If the suture runs toward the nerves, blood vessels, and internal organs, it is possible there will be a corresponding damage. Or it can run toward and pierce through the skin, like the case we reported here.

This case indicated potential risks that may exist in the application of the barbed sutures for deep tissue suturing. Design and manufacture of barbed sutures need to be improved in order to remove the risk and keep the advantages at the same time, such as remaining the main suture without breaking before the barbs are fully absorbed.

## Authorship

SL, HS, ZY, TW, and XY: performed the operation. SL, WW, and XY: wrote the manuscript.

## Conflict of Interest

No conflict of interests were declared by the authors.
